# Methylation Status of Corticotropin-Releasing Factor (CRF) Receptor Genes in Colorectal Cancer

**DOI:** 10.3390/jcm10122680

**Published:** 2021-06-18

**Authors:** Maria Panagopoulou, Antonia Cheretaki, Makrina Karaglani, Ioanna Balgkouranidou, Eirini Biziota, Kyriakos Amarantidis, Nikolaos Xenidis, Stylianos Kakolyris, Stavroula Baritaki, Ekaterini Chatzaki

**Affiliations:** 1 Laboratory of Pharmacology, Medical School, Democritus University of Thrace, GR-68100 Alexandroupolis, Greece; mpanagop@med.duth.gr (M.P.); antoniaheretaki@gmail.com (A.C.); makrina.karaglani@gmail.com (M.K.); ioannabio@yahoo.it (I.B.); 2 Department of Medical Oncology, Medical School, Democritus University of Thrace, GR-68100 Alexandroupolis, Greece; eirinibiziota@gmail.com (E.B.); kyriakos.amarantidis@gmail.com (K.A.); nxenidis@gmail.com (N.X.); skakolyr@med.duth.gr (S.K.); 3 Laboratory of Experimental Oncology, Division of Surgery, School of Medicine, University of Crete, GR-71003 Heraklion, Greece; 4 Hellenic Mediterranean University Research Centre, Institute of Agri-Food and Life Sciences, GR-71410 Heraklion, Greece

**Keywords:** CRF, colorectal cancer, methylation, bioinformatics, machine learning, liquid biopsy

## Abstract

The corticotropin-releasing factor (CRF) system has been strongly associated with gastrointestinal pathophysiology, including colorectal cancer (CRC). We previously showed that altered expression of CRF receptors (CRFRs) in the colon critically affects CRC progression and aggressiveness through regulation of colonic inflammation. Here, we aimed to assess the potential of *CRFR* methylation levels as putative biomarkers in CRC. In silico methylation analysis of CRF receptor 1 (*CRFR1*) and CRF receptor 2 (*CRFR2*) was performed using methylome data derived by CRC and Crohn’s disease (CD) tissues and CRC-derived circulating cell-free DNAs (ccfDNAs). In total, 32 and 33 differentially methylated sites of CpGs (DMCs) emerged in *CRFR1* and *CRFR2*, respectively, between healthy and diseased tissues. The methylation patterns were verified in patient-derived ccfDNA samples by qMSP and associated with clinicopathological characteristics. An automated machine learning (AutoML) technology was applied to ccfDNA samples for classification analysis. In silico analysis revealed increased methylation of both *CRFRs* in CRC tissue and ccfDNA-derived datasets. *CRFR1* hypermethylation was also noticed in gene body DMCs of CD patients. *CRFR1* hypermethylation was further validated in CRC adjuvant-derived ccfDNA samples, whereas *CRFR1* hypomethylation, observed in metastasis-derived ccfDNAs, was correlated to disease aggressiveness and adverse prognostic characteristics. AutoML analysis based on *CRFRs* methylation status revealed a three-feature high-performing biosignature for CRC diagnosis with an estimated AUC of 0.929. Monitoring of *CRFRs* methylation-based signature in CRC tissues and ccfDNAs may be of high diagnostic and prognostic significance in CRC.

## 1. Introduction

CRC is among the most common cancers worldwide, accounting for 10.2% of the newly diagnosed malignancies and 9.2% of cancer-related deaths in 2018 [1]. Although the five-year survival rates for early-stage CRC reach 90%, mortality is significantly high among patients with distant metastases [2]. Hence, tumor stage at the time of diagnosis is thought to be the most crucial prognostic factor. Early diagnosis is often delayed by lack of symptoms such as abdominal pain, rectal bleeding, weakness and weight loss which usually appear in late-stage CRC, accompanied by worse prognosis [3]. Therefore, preventive screening of asymptomatic individuals should be mandatory for reducing the incidence and mortality rates of CRC [4]. Currently, preventive colon cancer screening and early diagnosis are facilitated only by invasive methods such as colonoscopy and sigmoidoscopy. The success and accuracy of this method depend on several parameters, including the experience of the gastroenterologist and the correct implementation of the technical protocols, such as adequate bowel preparation and avoidance of bleeding complications [5,6]. In addition, randomized controlled trials have shown that sigmoidoscopy is limited in detecting cancers only in distal colon [7], while computed tomography (CT) colonography has shown lower sensitivity in detecting small polyps (<8 mm) [8]. Furthermore, radiation exposure and the need for additional colonoscopy in case of positive findings are also considerable disadvantages of the above methods [9]. The development of noninvasive approaches for early CRC screening has recently begun to gain ground. In this context, guaiac-based fecal occult blood test (gFOBT), a method that detects blood in feces, has demonstrated low sensitivity (51%) in detecting cancer cells [10], whereas DNA stool tests (i.e., Cologuard^®^) have high sensitivity for early CRC detection (92%) but lower performance in perceiving advanced pre-cancerous lesions (42.4%) [11]. Blood-based protein biomarkers have been suggested for the early detection of CRC [12]. Circulating cfDNA has been considered as a liquid biopsy material able to provide insights in cancer initiation and progression, meeting the need for a convenient, minimally invasive tool for precision medicine [13,14,15]. It is well-established that specific gene methylation changes that occur early in carcinogenesis and can be detected in ccfDNA could serve as valuable biomarkers for early cancer screening [16,17,18]. For example, a SEPT9 methylation test in ccfDNA has been introduced as a screening option for detecting CRC; however, it shows low rates of sensitivity (48%) and specificity (92%) [9]. Hence, the identification of novel biomarkers in liquid biopsy materials could be of great importance in early CRC screening.

Stress affects the function of the gastrointestinal tract in multiple ways and has been associated with its pathology [19,20]. The hypothalamic neuropeptide CRF and its homolog urocortin coordinate neuroendocrine pathways of the stress response via activation of two distinct receptors, CRFR1 and CRFR2. CRFRs are expressed throughout the gastrointestinal tract [19,20,21,22,23], while aberrant expression has been reported in CRC [24] and in Crohn’s disease [25,26,27,28] considered to be a high-risk premalignant condition. In CRC, expression of CRFR1 [29] and decreased expression of CRFR2 [30] are associated with tumorigenicity and progression, whereas the CRF system has been implicated in cancer development in multiple tissues [31].

Although the expression of CRFRs is known to be regulated by methylation in the central nervous system (CNS) [32,33], the methylation status of the CRFRs present in peripheral sites has not been thoroughly studied. We recently reported on an association of *CRFR1* hypermethylation with the presence of steroid hormone receptors in breast cancer [34]. In addition, using genome-wide methylation approaches, Kobayashi et al. found that hypermethylation of *CRFR2* is correlated with colitis-induced CRC [35]. This is in line with our previous reports indicating that diminished or lost CRFR2 expression in CRC promotes tumor development and aggressiveness, including tumor immunoescape, by regulating molecular circuits involved in sustaining inflammation in the gut [30,36]. Here, we explored the methylation status of CRFR1 and CRFR2 by performing bioinformatic analysis in DMCs identified in *CRFR1* and *CRFR2* genes obtained by publicly available high-throughput methylome data from healthy, CD and CRC colon tissue. We further examined the CRFRs methylation in patient-derived liquid biopsy samples (ccfDNA) and evaluated its diagnostic and prognostic value in CRC.

## 2. Materials and Methods

### 2.1. Dataset for the In Silico Analysis

Raw DNA methylation data from CRC, CD patients and normal colons as well as the corresponding clinicopathological parameters were obtained from the GEO (Gene Expression Omnibus) (https://www.ncbi.nlm.nih.gov/geo/, accessed on 1 September 2020) database [37]. CRC, CD and ccfDNA were used as keywords in the GEO query and ‘Methylation profiling by array’ as the study type. Thirty-four studies were found; of them, only those using Infinium Human Methylation 450K and EPIC BeadChips and providing adequate raw and clinical data were selected for further analysis. Four studies, namely GSE99788 [38], GSE149282 [39], GSE122126 [40] and GSE105798 [41], were recruited for our analysis. The description of study groups and correlations is presented in Table 1.

### 2.2. Data Preprocessing and DNA Methylation Analysis

Raw DNA methylation data (IDAT files) and sample annotation files were processed in the Bioconductor R package RnBeads v2.0 (https://rnbeads.org/index.html, accessed on 18 September 2020) [42]. CpGs were chosen as the genomic region of interest. The MethyLumi-Noob method was used to normalize technical variation in the background fluorescence signal [43]. BMIQ was used as a normalization method to adjust beta values of type II design probes into a statistical distribution characteristic of type I probes [44]. Probes for SNPs or probes outside of the CpG context as well as probes on sex chromosomes were removed [45]. Probes/samples with the highest fraction of unreliable measurements were removed from further analysis using the Greedycut algorithm. Νormalized β-values for each CpG were generated, representing the methylated probe’s intensity divided by the overall intensity (sum of methylated and unmethylated probe intensities) plus an offset of 100 [46]. DNA methylation differences were analyzed using hierarchical linear models implemented in the limma package [47] provided in the RnBeads pipeline. Differentially methylated CpGs (DMCs) for *CRFR1* and *CRFR2* were identified based on the false discovery rate (FDR-adjusted *p*-value < 5.00 × 10^−2^). 

### 2.3. In Silico Determination of Transcription Factor (TF) Binding

In order to examine if DMCs identified were correlated to *CRFR1* and *CRFR2* gene expression, we further analyzed promoter regions to locate TFs binding sites. First, the Methprimer (https://www.urogene.org/methprimer/, accessed on 5 November 2020) [48] tool was used for the identification of possible cytosine–guanine dinucleotides islands (CGIs) at these regions. CGIs are regions of transcription initiation with a high frequency of CpG sites. The criteria for CGIs prediction used were as follows: size region of at least 100 bp, GC percentage greater than 50% and an observed-to-expected CpG ratio greater than 60%. Then, the PROMO (http://alggen.lsi.upc.es/, accessed on 5 November 2020) [49] tool was used in order to define possible TFs binding in identified CGIs. Only human factors and human sites were considered for TFs search.

### 2.4. Clinical Samples

CRC patients who visited the Department of Medical Oncology of University General Hospital of Alexandroupolis between 2001 and 2016 were included in the study. Blood samples were collected following diagnosis from two patient groups: (a) 42 patients having undergone surgery for primary CRC within the previous month and before the initiation of adjuvant therapy (adjuvant group) and (b) 71 patients with metastatic disease following palliative surgery and before the initiation of first-line chemotherapy (metastatic group). The treatment modules used for the metastatic group were FOLFIRI (a combination of folinic acid, fluorouracil and irinotecan) or FOLFOX (a combination of folinic acid, fluorouracil and oxaliplatin) or the alternatives XELIRI (irinotecan and capecitabine) or XELOX (capecitabine and oxaliplatin) plus bevacizumab or panitumumab/cetuximab. The response of metastatic patients to the above regimens was correlated with *CRFR1* and *CRFR2* methylation. Clinicopathological and demographic characteristics are presented in Table 2. Follow-up data from 2001 to 2016 were also available. In parallel, blood samples from 20 healthy donors were collected from the blood donation unit of the same hospital and included in our study (11 males and nine females, mean age: 58.9 (± 9.0), median: 59.0 (range: 43.0–76.0), mean BMI: 26.0 (± 5.0), median: 24.5 (20.49–35.49)) (control group). Inclusion criteria of both patients and healthy individuals were age between 18 and 80 years old and the ability to give informed consent, for patients not to have initiated adjuvant or first-line treatment before sample collection and for healthy individuals to be free of cancer and cancer history. Peripheral blood was collected in an EDTA before treatment and processed immediately for plasma isolation within 2 h. The study was approved by the Scientific Board and the Ethics Committee of the University General Hospital of Alexandroupolis/Greece and was conducted according to the ethical principles of the Declaration of Helsinki. All the patients participated after signing a voluntary informed consent.

### 2.5. Extraction of ccfDNA

Circulating cfDNA was extracted from plasma using a MagCore Nucleic Acid Extraction Kit (RBC BIOSCIENCE, New Taipei City, Taiwan) and a MagCore^®^ Compact Automated Nucleic Acid Extractor (RBC BIOSCIENCE, New Taipei City, Taiwan) according to the manufacturer’s instructions. Briefly, ccfDNA was eluted from 1200 μL of plasma in 40 μL elution buffer and stored at −20 °C until further use.

### 2.6. Sodium Bisulfite Conversion of ccfDNA

Bisulfite conversion was performed by EZ DNA Methylation-Gold™ Kit (ZYMO Research Co., Orange, CA, USA) according to the manufacturer’s instructions and previous reports [50]. During conversion, all unmethylated but not the methylated cytosines of ccfDNA were converted to uracil. DNA was then eluted in 10 μL elution buffer and stored at −80 °C until use. In each experiment, CpGenome Human Methylated and Non-Methylated DNA standards (Merck Millipore, Germany) or H_2_O were included as positive and negative controls, respectively.

### 2.7. Quantitative Methylation-Specific PCR (qMSP)

Promoter methylation of *CRFR1* and *CRFR2* was analyzed by qMSP. A methylation-independent assay with non-CpG bearing sites for the β-actin gene (ACTB) was used in order to verify DNA quality and normalize results. Primer sequences and qMSP conditions are presented in detail in our previous work [34]. The samples were run in duplicates. The results were calculated using the Rotor-Gene 6000 Series Software 1.7 (Qiagen). The results were analyzed using the 2^−^^ΔΔCT^ formula representing methylation levels, where ΔΔCT = ΔCTsample – ΔCTcalibrator [51]. Negative and positive control samples of 0% and 100% methylated converted DNA standards were included in each run.

### 2.8. Statistical Analysis

The Kolmogorov–Smirnov test was applied to check for normality in distribution and the chi-squared test for comparison between discrete variables. One-way ANOVA test followed by Bonferroni post-hoc or Kruskal–Wallis test were used for comparisons of continuous variables between three or more subgroups. In case of binary variables, *t*-test or Mann–Whitney test were also applied. Pearson or Spearman correlation was applied to compare two continuous variables. Metastatic patients who presented complete response (CR), partial response (PR) to treatment or stable disease (SD) at the first clinical examination after first-line treatment initiation according to response evaluation criteria in solid tumors (RESIST) version 1.1 [52] were considered as “responders,” whereas those who presented clinical progressive disease (PD) were considered as “non-responders.” Statistical significance was set at *p*-value < 0.05. Statistical analysis was performed using the IBM SPSS 19.0 statistical software (IBM Corp., 2010, IBM SPSS Statistics for Windows, version 19.0, Armonk, NY, USA).

### 2.9. Automated Machine Learning Analysis

Our data were further analyzed using an AutoML technology in order to construct signatures of diagnostic value, combining the liquid biopsy-based experimental parameters determined by our study and the clinicopathological features of the study groups. The Just Add Data Bio v1.1.118 (JADBio) (https://www.jadbio.com/, accessed on 25 January 2020) platform applicable to low-sample high-dimensional datasets [53] was employed and able to provide predictive models by employing standard, best-practice and state-of-the-art statistical and machine learning methods. JADBio works as follows: it first selects the appropriate algorithms to try for the task at hand depending on the outcome type, predictor type, user preferences (e.g., importance of quality of analysis vs. speed of analysis) using an artificial intelligence decision support system. The algorithms are selected to perform the following steps: data transformations, data preprocessing and imputation of missing values, feature selection, predictive modeling and data visualization. The AI system also selects which tuning hyperparameter values to try for each algorithm. All combinations of algorithms for each step and hyperparameter values (called configurations) are applied using a 10-fold cross-validation protocol. JADBio applies a bootstrap-based adjustment to the final reported performance [54] to remove this optimism and to return slightly conservative estimates of performance. JADBio performs biosignature discovery using SES (statistical equivalent signature) or LASSO (least absolute shrinkage and selection operator) algorithms for feature selection. A signature is defined as a minimal-size subset of predictors (features, molecular quantities, biomarkers, risk factors), which collectively (multivariately) lead to an optimal predictive model, neglecting all other features as irrelevant or redundant for prediction given the selected features. For classification modeling, JADBio employs an SVM (support-vector machine) [55] with full polynomial and Gaussian kernels, random forests [56], ridge logistic regression [57] and decision trees [58]. As most modern machine learning models are completely incomprehensible to a human, JADBio reports not only the best-out-of-all model, but also the best interpretable model (linear models or decision trees).

## 3. Results

### 3.1. In Silico Analysis of CRFR1 and CRFR2 Methylation in CRC and CD

In silico methylation analysis of *CRFR1* and *CRFR2* genes was performed using methylome data derived by CRC and CD tissues and CRC ccfDNAs. The results are described below.

#### 3.1.1. Analysis in CRC and CD Tissue-Derived Datasets

For the methylation analysis of the indicated receptor genes in CRC and CD tissues, we used methylome data from four datasets (GSE149282, GSE122126, GSE105798, GSE99788) (Table 1). DMCs were identified among groups in all the studies (Supplementary Tables S1 and S2) except for GSE99788. In total, 32 and 33 DMCs were detected among healthy individuals and CD or CRC patients for *CRFR1* and *CRFR2,* respectively. In general, lower methylation was noticed mostly in gene body intronic regions, while higher methylation was observed mainly in the first exon and the TSS200/1500 genome locations, known to be strongly associated with regulation of expression by methylation [59,60]. We therefore focused our analysis mostly in those regions. Among the DMCs identified in *CRFR1,* four CpGs (cg08473090, cg08929103, cg12577105 and cg18757974) were located at TSS1550 and two (cg11338426, cg13521908)—at the first exon (Table 3). Notably, methylation in all the CpGs located in the island region, known to have an important role in transcriptional regulation [61], was increased in CRC tissue in comparison to the adjacent normal tissue (GSE149282 dataset), whereas decreased methylation was noticed in the one found in the N shore, a region 0–2 kb upstream (5′) of the CpG island. Other *CRFR1*-related DMCs identified in CRC or CD tissue databases were located in the gene body or in the 5′UTR. A decrease of methylation was mainly noticed at these DMCs, while an increase was detected at DMCs located at islands or S shores of the gene body or the 5′UTR in cancer tissue in relation to the adjacent healthy colon (GSE149282 and GSE1222126). An increase of *CRFR1* methylation was also noticed in gene-body DMCs of CD patients in comparison to normal tissues (GSE105798) (Supplementary Table S1). Finally, only one DMC (cg21773872) emerged from the GSE105798 dataset, showing decreased methylation in CD in relation to normal colon tissues.

For *CRFR2*, nine CpGs located close to the TSS (cg01718447, cg02712145, cg04863452, cg07658503 cg13094036, cg14896516, cg15615793, cg18351440, cg21773872) and six CpGs located at the first exon (cg04922810, cg04923928, cg18266052, cg24214442, cg24610236, cg27430726) were identified as DMCs among the studied groups (Table 4). They were all found in the island except one found in the S shore (a region 0–2 kb downstream (3′) of the CpG island) and one in the N shelf (a region 2–4 kb upstream (5′) of the CpG island). In the GSE149282 dataset, all of the 11 CpGs (cg01718447, cg02712145, cg07658503, cg13094036, cg14896516, cg21773872, cg04922810, cg18266052, cg24214442, cg24610236, cg27430726) identified as DMCs were hypermethylated in cancer tissue in relation to the adjacent healthy colon.

#### 3.1.2. Analysis of CRC-Derived ccfDNA Data

Two CpGs (cg08929103, cg13521908) identified in *CRFR1* as DMCs in ccfDNA dataset GSE1222126 showed the same hypermethylation trend in patient-derived samples compared to normal counterparts, thus suggesting that ccfDNA may reflect the methylation status of tumor tissues. Methylation analysis of *CRFR2* using the same dataset revealed that three CpGs (cg04863452, cg15615793, cg04923928) and one CpG (cg18351440, located in the N shelf) were hypermethylated and hypomethylated, respectively, in CRC ccfDNAs compared to their normal counterparts. It has to be noted that the DMCs found in CRC ccfDNA (GSE1222126 dataset) were not identified as DMCs in the CRC tissue (GSE149282).

### 3.2. In Silico Analysis of TFs Binding in CRFR1 and CRFR2 Promoters

DNA methylation regulates gene expression mainly by disturbing transcription factor (TF) and RNA polymerase binding to putative sites known to be necessary for initiation of transcription [62]. To address whether the identified DMCs may actually play a role in regulating CRFR1 and CRFR2 expression, we examined their presence within CGIs that are known to contain TFs binding that could initiate transcription.

**Figure 1:**
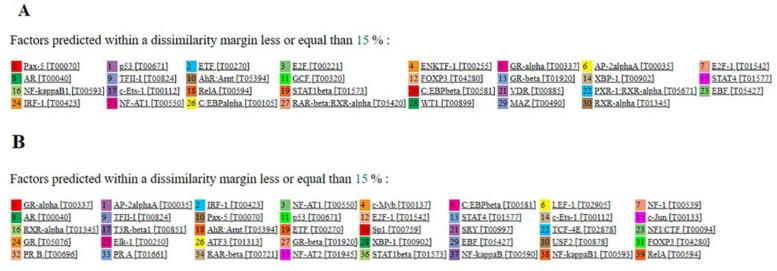
Predicted TFs for binding to the CGIs of *CRFR1* and *CRFR2* promoters containing DMCs: (**A**) 31 TFs were predicted for *CRFR1* and (**B**) 40 TFs were predicted for *CRFR2* with a dissimilarity margin ≤ 15%. TFs: transcription factors; CGIs: CpG islands; CRFR1: human corticotropin-releasing factor receptor 1; CRFR2: human corticotropin-releasing factor receptor 2.

For *CRFR1,* one CGI of 180 bp was found that contained DMCs between malignant and adjacent colon tissues. Further analysis by the PROMO tool predicted 31 putative TFs that could bind to this CGI (Figure 1A). For *CRFR2*, a CGI of 287 bp was also identified and 40 TFs were predicted (Figure 1B). Together, these findings show that CGIs containing the identified DMCs contain multiple sites for putative TFs binding and therefore altered methylation during carcinogenesis can affect *CRFR1* and *CRFR2* expression.

### 3.3. Methylation Analysis of CRFR1 and CRFR2 in CRC-Derived ccfDNA Clinical Samples

Following the in silico analysis, methylation of the CRF receptor genes was investigated in CRC patient-derived ccfDNAs and compared with their healthy counterparts. Quantitative MSP assays were performed in ccfDNAs isolated from 42 adjuvant CRC patients, 71 metastatic CRC patients and 20 healthy individuals (control). Primers of *CRFR1* and *CRFR2* were designed at the promoter region inside the studied CGI.

**Figure 2:**
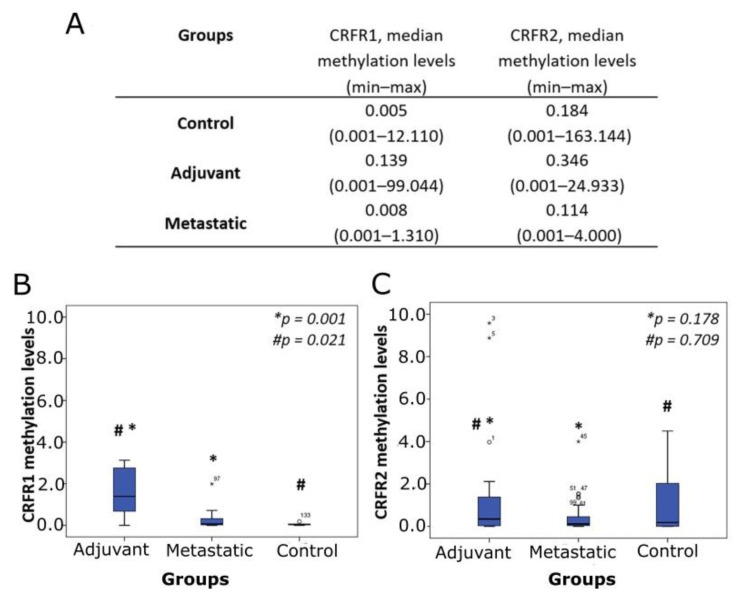
Methylation levels of CRF receptor genes estimated by qMSP in ccfDNA from adjuvant and metastatic CRC patients and healthy individuals (control). (**A**) Median methylation levels of CRFR1 and CRFR2. Boxplots depict methylation levels in relation to study groups for (**B**) *CRFR1* and (**C**) *CRFR2*; * p: adjuvant vs. metastatic; *# p*: adjuvant vs. control; ccfDNA: circulating cell-free DNA; qMSP: quantitative methylation-specific PCR; CRC: colorectal cancer.

*CRFR1* methylation was detected in 45.0, 35.7 and 36.6% of control, adjuvant and metastatic groups, respectively. For *CRFR2*, the respective numbers were 70.0, 64.3 and 67.6%. CRF receptor methylation levels are presented in Figure 2A. Significantly increased levels of *CRFR1* methylation were found in the adjuvant group compared to the control group (*p* = 0.021) and the metastatic group (*p* = 0.001) (Figure 2B). For *CRFR2*, no statistically significant differences in methylation levels were observed between the studied groups (Figure 2C).

**Figure 3:**
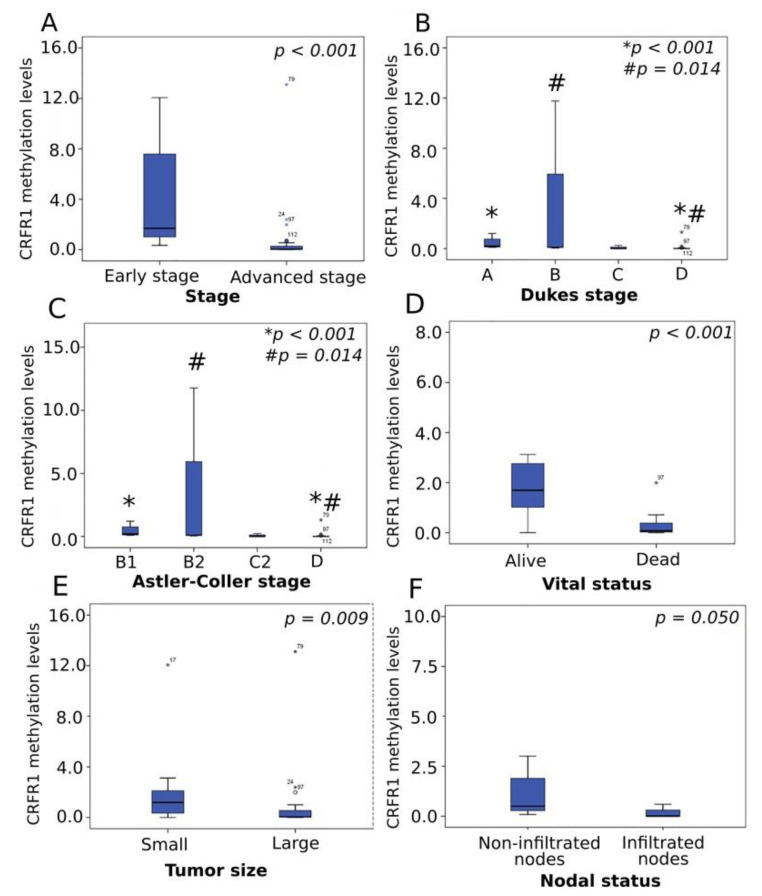
Correlation of CRFR1 methylation levels with clinical parameters. Boxplots depict *CRFR1* methylation levels in relation to (**A**) stage, (**B**) Dukes stage, (**C**) Astler–Coller stage, (**D**) vital status, (**E**) tumor size and (**F**) nodal status; * *p:* A vs. D for Dukes stage and B1 vs. D for Astler–Coller stage; # *p:* B vs. D for Dukes stage and B2 vs. D for Astler–Coller stage.

Interestingly, among cancer patients, lower methylation levels of *CRFR1* were correlated with adverse clinicopathological characteristics and poor outcomes. Specifically, low methylation levels were significantly correlated with advanced disease stage (*p* < 0.001) (Figure 3A), D tumor stage (by Dukes) as compared to A (*p* < 0.001) and B (*p* = 0.014) stages (Figure 3B), while the same methylation trend was observed in the D stage when compared to B1 (*p* < 0.001) and B2 (*p* = 0.014) stages of the Astler–Coller classification system (Figure 3C).

Diminished *CRFR1* methylation was also associated with the incidence of death (*p* < 0.001) (Figure 3D). Moreover, within the adjuvant group, larger tumors and those presenting lymph node infiltration showed decreased *CRFR1* methylation levels in relation to small tumors (*p* = 0.009) (Figure 3E) and those without characteristics of LN infiltration (*p* = 0.050) (Figure 3F), respectively. No significant correlations were established between *CRFR1* methylation levels and tumor location, grade, mutational status of the KRAS gene or demographic parameters. Similarly, *CRFR1* methylation levels were not associated with the frequency of relapses in the adjuvant group or first-line treatment response in the metastatic group. *CRFR2* methylation levels were not associated with any clinicopathological parameters or prognosis in a statistically significant manner. The methylation analysis of the ccfDNA study groups revealed that *CRFR1* methylation was significantly increased in the adjuvant group of patients compared to that of healthy individuals and patients with metastatic disease (Figure 2B). Comparison of the *CRFR1* methylation levels between the last two groups (control and metastatic) also showed a trend of hypermethylation in the metastatic group; however, this difference was not statistically significant. Within the patients’ groups (adjuvant and metastatic), the lower methylation levels were positively correlated with advanced tumor stage and size, infiltrated nodes and poor outcomes (Figure 3A–F). Furthermore, low methylation within the two malignant groups was significantly associated with advanced disease and adverse prognosis. The above findings demonstrate for the first time *CRFR1* hypermethylation as a hallmark of early CRC status, which is remarkably diminished as the disease progresses to more aggressive phenotypes, reaching the almost normal levels.

### 3.4. Automated Machine Learning Analysis

**Figure 4:**
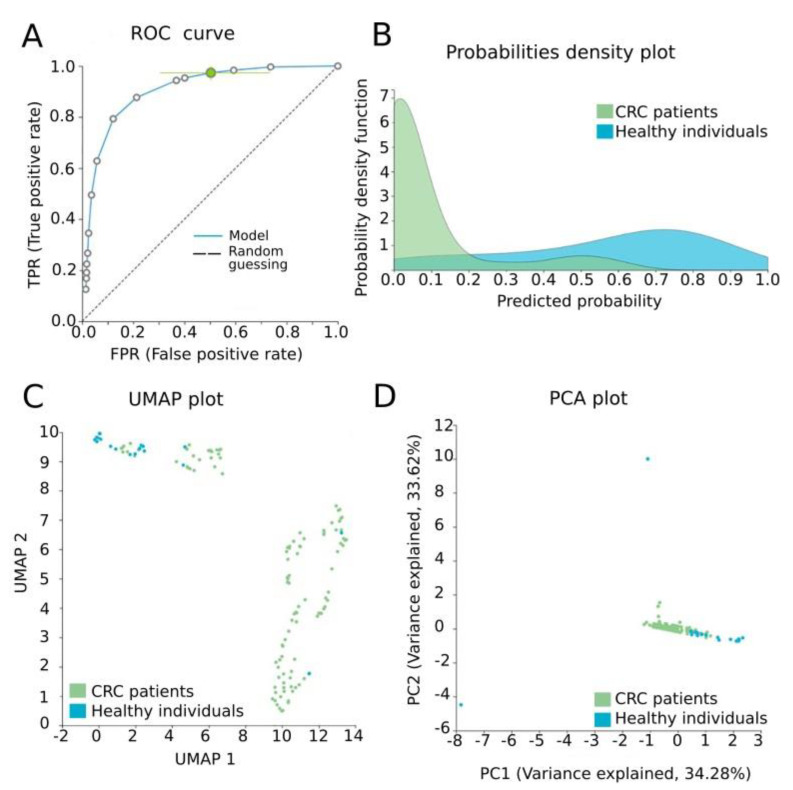
Model performance for the discrimination of CRC patients from healthy individuals. (**A**) ROC curve of the model, AUC: 0.929 (0.873, 0.972). (**B**) Probabilities density plot depicting distributions between normal (class 0, green) and CRC ccfDNA samples (class 1, blue). (**C**) UMAP plot showing sufficient discrimination between normal (class 0, blue) and CRC ccfDNA samples (class 1, green). (**D**) PCA plot presenting a good separation between normal (class 0, blue) and CRC ccfDNA samples (class 1, green). ROC: receiver operating characteristic; PCA: principal component analysis; UMAP: uniform manifold approximation and projection.

**Figure 5:**
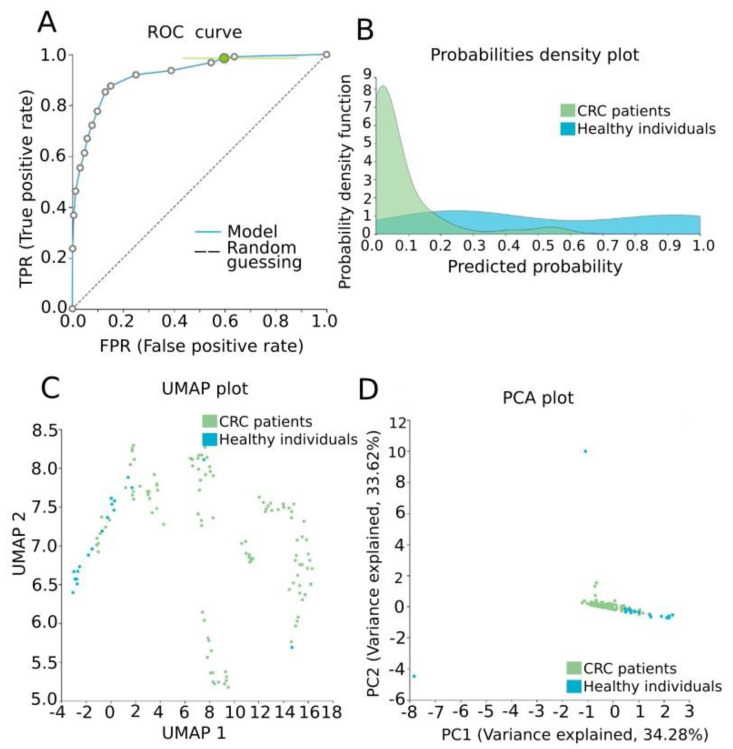
Model performance for the discrimination of CRC patients from healthy individuals. (**A**) ROC curve of the model, 0.933 (0.887, 0.972). (**B**) Probabilities density plot depicting distributions between normal (class 0, green) and CRC ccfDNA samples (class 1, blue). (**C**) UMAP plot showing good discrimination between normal (class 0, blue) and CRC ccfDNA samples (class 1, green). (**D**) PCA plot presenting a good separation between normal (class 0, blue) and CRC ccfDNA samples (class 1, green).

Our data were further analyzed by AutoML in order to construct a signature of diagnostic value, combining methylation measurements and established clinicopathological features of the study group. The task was a classification analysis in order to discriminate CRC patients from healthy individuals. Our best-performing model was a three-feature signature containing the methylation levels of *CRFR1*, the methylation levels of *CRFR2* and age via the Classification Random Forests algorithm with an estimated area under the curve (AUC) of 0.929 (0.873, 0.972) and an average precision of 0.983 (0.965, 0.994) to discriminate healthy tissues from CRC. Model performance and model inspection are depicted in Figure 4. Furthermore, the best-interpretable model containing the same features via ridge logistic regression was produced reaching an AUC of 0.933 (0.887, 0.972) and an average precision of 0.983 (0.965, 0.994) (Figure 5).

## 4. Discussion

We and others have reported that CRF receptors play a critical role in regulating inflammation, carcinogenesis and disease progression in the colon [24]. Although the epigenetic regulation of CRF receptors has been thoroughly studied in stress-related CNS disorders, their methylation patterns and role in peripheral tissues have not been clearly elucidated. In this study, we first adopted a bioinformatic approach using publicly available datasets of CRC and CD to analyze methylation of CRF receptor genes. Although a large number of DMCs was identified in the entire genes (32 and 33 in *CRFR1* and *CRFR2,* respectively) among healthy tissues and studied pathologies, we focused our analysis on the first exon and the TSS200/1500 region, which are genome locations known to be strongly associated with gene expression regulation by methylation [59,60].

Methylation of CRFR1 CpGs within the above regions and in particular those located in islands was significantly elevated in CRC compared to the adjacent healthy colon tissues, thus pointing to downregulation of CRFR1 expression. This pattern of *CRFR1* hypermethylation was also observed in methylome datasets of ccfDNAs from CRC patients and clinical ccfDNA samples obtained in adjuvant CRC cases. Taken together, these findings suggest that assessing methylation in ccfDNA can dynamically reflect methylation events in the tumor’s lifespan. This notion is further supported by recent reports in pancreatic cancer, demonstrating that DNA methylation profiles of cancerous tissues and respective ccfDNAs significantly correlate with each other [38]. In concordance, our results for CRC ccfDNAs showed that methylation levels of *CRFR1* were increased in the adjuvant group of patients in relation to healthy individuals. However, within the patients’ groups, lower methylation levels were correlated with advanced tumor stage and size, infiltrated nodes and poor outcomes. It can be postulated that *CRFR1* methylation is a molecular event characterizing primary tumor formation and is later lost during transition to the metastatic phase. Our findings are the first demonstrating aberrant *CRFR1* methylation in CRC. Given that decreased methylation may lead to increased CRFR1 expression, our findings strongly support an involvement of CRFR1 signaling in CRC aggressiveness, as previously shown in mouse models where CRFR1 showed a proinflammatory and a pro-tumorigenic effect in inflammation-related colon cancer [63]. In this context, a CRFR1-mediated CRC promoting pathway has also been described in CRC cells through modulation of IL-6/JAK2/STAT3 signaling and VEGF-induced angiogenesis [29], while *CRFR1* hypermethylation in breast cancer models has been associated with the expression of steroid hormone receptors, a favorable prognostic factor [34]. Overall, our findings are quite novel, given that this is the first study reporting aberrant *CRFR1* methylation in CRC, as per our knowledge.

Although we showed increased CRFR1 methylation levels in the adjuvant group compared to healthy individuals and metastatic group, we failed to establish any significant alterations in the methylation levels assessed in the control and metastatic study groups. At the same time, CRFR1 hypomethylation within the cancer groups (adjuvant and metastatic) revealed direct clinical relevance as it was positively correlated with adverse clinicopathological characteristics and poor outcomes. Based on the findings extrapolated by the two cancer groups, one could postulate that CRFR1 methylation might be a molecular event characterizing primary tumor onset which is significantly eliminated later, during tumor transition to a metastatic phase. Given the limitations of our in silico analysis in terms of the available patient study groups, we can further speculate that this trend of CRFR1 hypermethylation followed by hypomethylation as the disease progresses may be associated with the sample origin and thus be ccfDNA-specific. Our findings are novel, demonstrating that primary CRC is characterized by aberrant CRFR1 methylation which is progressively lost following disease aggressiveness and therefore the CRFR1 methylation levels may have a prognostic significance in CRC.

In silico analysis of *CRFR2* methylation status revealed that all the emerged DMCs identified in transcription-related regions were hypermethylated in the CRC tissues compared to the healthy ones. This is in line with recent findings indicating positive correlation of hypermethylated *CRFR2* (studied by an Infinium Human Methylation 450K array) with colitis-induced CRC [35]. Accordingly, the previously reported observations on diminished CRFR2 mRNA and protein expression in CRC cell lines and tissues [30] may be attributed to *CRFR2* hypermethylation. We have further identified the CRFR2/Ucn2 signaling as a critical negative regulator in sustaining chronic inflammation and promoting cancer development and aggressiveness in the colon [24]. Based on the above findings we strongly suggest that *CRFR2* methylation and expression levels in the colon tissues may be of prognostic significance in CRC management. In the CRC ccfDNA dataset, three more CpGs of CRFR2 were found hypermethylated. However, in our experimental part of the study, no statistically significant differences in CRFR2 methylation levels were observed between healthy and patient-derived ccfDNAs analyzed by qMSP, raising doubts about the clinical application in liquid biopsy.

Furthermore, methylation analysis in gene body intronic regions of *CRFR1* and *CRFR2* revealed lower methylation levels in both receptors in CRC compared to normal tissues. Gene body methylation is largely unexplored and very often connected to active transcription [64,65], which is known as the DNA methylation paradox [66]. Although gene body hypomethylation has been previously correlated to cancer [67], further targeted experimental analysis of gene body *CRFR1* and *CRFR2* methylation in relation to expression may be necessary in order to clarify their interconnection in our system.

Epigenetic modifications such as DNA methylation within CGIs are known to modulate the dynamic binding of transcription factors to regulatory elements, thus resulting in transcriptional repression [68]. In our study, we predicted 31 and 40 TFs that may regulate *CRFR1* and *CRFR2* transcription, respectively, through putative binding to DMC-containing CGIs. Therefore, any methylation events in these DMCs during malignant transformation may block TFs binding and transcriptional activities. Among the predicted TFs, NF kappa B, AP-2 and E2F have been previously shown to be sensitive to CpG methylation with consequent inhibition of their DNA binding activities [62]. In addition, p53 was also predicted, and CRF has been reported to inhibit cell proliferation and apoptosis in cell lines via the CRFR1-mediated p53 mechanism [69].

To further exploit our experimental observations and bring in a clinical perspective, we employed an autoML approach in order to build classifying models of diagnostic/prognostic performance. ML exploits a variety of algorithms to perform predictive analysis and its use in biomarker discovery in cancer is rapidly increasing [70,71]. Automated tools for ML promise to democratize data analysis for non-experts, increase productivity, improve replicability of the statistical analysis and shield against common methodological analysis errors such as overfitting [53]. We employed JADBio, an autoML platform designed for standard, best-practice and state-of-the-art statistical and machine learning methods. JADBio has previously been successfully used to produce signatures for clinical applications such as development of classifiers for metastatic BrCa based on novel ccfDNA methylation patterns [16], identification of risk of lung cancer in smokers [72] or suicide prediction among depressive patients [73]. Recently, by revisiting publicly available omics datasets via JADBio, we were able to deliver accurate highly-performing blood-based predictive biosignatures in Alzheimer’s disease [74] and in breast cancer [75].

In our study, JADBio analysis delivered a three-feature biosignature via the Classification Random Forests algorithm, including both CRF receptor methylation levels assessed in ccfDNA and age, with high-performance metrics in discriminating between CRC patients and healthy individuals. It has to be noted that although methylation of *CRFR2* did not reach a statistically significant difference between groups when examined by standard univariate statistical analysis, multivariate analysis by JADBio selected it as a significant feature when combined with *CRFR1* methylation and age. Previous ML-based studies performed in ccfDNA materials have also shown promising results in CRC. Wan et al. built an ML-based model for the early detection of CRC with an AUC of 0.92 (95% CI: 0.91–0.93) [76], while Luo et al. constructed a predictive model that accurately discriminated CRC patients from healthy individuals (AUC: 0.96) [77].

Overall, the identification of novel, noninvasive and high-performance biomarkers is of high importance for early, pre-asymptomatic CRC diagnosis. In this context, we strongly believe that our novel three-feature biosignature could potentially have a clinical application in early CRC screening. Upon perspective clinical validation, our model, built on qMSP rather than whole-genome deep-sequencing methodologies, may offer a feasible liquid biopsy-based solution for early CRC diagnosis that can be implemented in any standard molecular diagnostic laboratory. It is important to mention that for successful clinical implementation, a standard operational procedure for ccfDNA preanalytical preparation should be adopted between labs. However, limitations of our study, including the small number of participants in the available patient study groups, may be responsible for restricting putative correlations between *CRFR1* and *CRFR2* methylation levels and clinicopathological features such as tumor stage and metastasis. For future clinical implications, our preclinical model’s performance needs to be validated in a larger independent group of patients and confirmed in a prospective clinical study, which is the greatest challenge in translating such findings into clinical practice. Furthermore, studies employing in vitro models should clarify the exact timing of methylation events during the malignant transformation process and their results in receptor protein expression. Percentages of methylation detection in patient groups are a clear finding of our study without, however, some statistical significance. Based on our methodology, samples showing no methylation are unmethylated rather than unsuitable for methylation detection due to low sample quality or abundance. Given that ccfDNA is a valuable source for tracing molecular alterations of a tumor, detected changes in the methylation profile of important genes could reflect a dynamic tumor burden. Our results show that although the detection of *CRFR1* and *CRFR2* methylation in ccfDNA does not hold a diagnostic value, methylation levels considered in the context of an ML-built biosignature can help in differentiating healthy individuals from CRC patients.

## 5. Conclusions

CRC is one of the most lethal cancers due to a long asymptomatic phase and difficulties in diagnosis, prognosis and disease management. Our presented data introduce the methylation levels of both CRFR1 and CRFR2 as putative biomarkers in CRC based on our novel biosignature. The prognostic significance of the CRFR1 methylation status is further supported by the findings showing that decreased ccfDNA CRFR1 methylation levels among CRC patients are correlated with tumor aggressiveness and poor clinical outcomes. Along with our previously reported findings [20,26], we suggest here that the CRFR2 hypermethylation patterns in CRC determined by bioinformatic analysis may be associated with decreased receptor expression, which in turn contributes to CRC progression and metastatic potential in accordance with our previous findings. In addition, we demonstrate that the CRFR1 methylation levels assessed in liquid biopsy could offer a minimally invasive approach, overcoming current obstacles in CRC tissue methylation monitoring. To this end, a three-feature biosignature was built, which can help ensure accurate disease diagnosis upon prospective evaluation.

## Figures and Tables

**Table 1:** Methylome datasets used for the bioinformatic analysis of *CRFR1* and *CRFR2* methylation.

Dataset	Platform	Correlated Groups	References
GSE149282	EPIC	Twelve CRC vs. 12 adjacent colon tissues	[35]
GSE122126	450k	Four CRC ccfDNAs vs. four healthy ccfDNA	[36]
GSE105798	450k	Three CD vs. eight normal colon tissues	[37]
GSE99788	EPIC	Thirteen CD vs. five normal colon fibroblasts	[34]

CRC: colorectal cancer; CD: Crohn’s disease; NINF: noninflammatory; STEN: stenotic; INF: inflammatory.

**Table 2:** Demographic and clinicopathological characteristics of CRC patient and normal groups.

Clinical Parameter	Total (*n* = 113) (%)	Adjuvant Group (*n* = 42) (%)	Metastatic Group (*n* = 71) (%)	Normal (*n* = 20) (%)
**Age (years)**				
Mean ± SD	67.0 ± 9.7	69.5 ± 8.7	65.5 ± 10	58.9 (±9.0)
Median, range	68, 44–87	70.5, 47–87	67, 44–85	59, 43–76
**Gender**				
Male	78 (69%)	30 (71.4%)	48 (67.6%)	11 (55%)
Female	35 (31%)	12 (28.6%)	23 (32.4%)	9 (45%)
**BMI**				
<18.5	2 (1.8%)	0	2 (2.8%)	0
18.5–24.9	12 (10.6%)	6 (14.3%)	6 (8.5%)	8 (40%)
25–29.9	35 (31%)	24 (57.1%)	11 (15.5%)	6 (30%)
≥30	13 (11.5%)	11 (26.2%)	2 (2.8%)	3 (15%)
Not available	51 (45.1%)	1 (2.4%)	50 (70.4%)	3 (15%)
**Cancer location**				
R	19 (16.8%)	13 (31%)	6 (8.5%)	
A	7 (6.2%)	4 (9.5%)	3 (4.2%)	
S	28 (24.8%)	18 (42.9%)	10 (14.1%)	
T	2 (1.8%)	2 (4.8%)	0	
D	3 (2.7%)	3 (7.1%)	0	
C	4 (3.5%)	2 (4.8%)	2 (2.8%)	
Νot available	50 (44.2%)	0	50 (70.4%)	
**Dukes classification**				
A	14 (12.4%)	14 (33.3%)	0	
B	14 (12.4%)	13 (31%)	0	
C	13 (11.5%)	13 (31%)	0	
D	69 (61%)	0	71 (100%)	
Not available	3 (2.7%)	2 (4.8%)	0	
**Astler–Coller classification**				
A	2 (1.8%)	2 (4.8%)	0	
B1	12 (10.6%)	12 (28.6%)	0	
B2	13 (11.5%)	13 (31%)	0	
B3	0	0	0	
C1	1 (0.9%)	1 (2.4%)	0	
C2	12 (10.6%)	12 (28.6%)	0	
C3	0	0	0	
D	71 (62.8%)	0	71(100%)	
Not available	2 (1.8%)	2 (4.8%)	0	
**Stage**				
Ι	15 (13.3%)	15 (35.7%)	0	
ΙΙ	13 (11.5%)	13 (31%)	0	
ΙΙΙ	13 (11.5%)	13 (31%)	0	
IV	71 (62.8%)	0	71(100%)	
Not available	1 (0.9%)	1 (2.4%)	0	
**Grade**				
1	41 (36.3%)	18 (42.9%)	14 (19.7%)	
2	50 (44.2%)	18 (42.9%)	41 (57.7%)	
3	12 (10.6%)	5 (11.9%)	7 (9.9%)	
Not available	10 (8.9%)	1 (2.4%)	9 (12.7%)	
**Tumor size**				
T1	3 (2.7%)	3 (7.15%)	0	
T2	24 (21.2%)	13 (30.95%)	11 (15.5%)	
T3	66 (58.4%)	21 (50%)	45 (63.4%)	
T4	12 (10.6%)	4 (9.5%)	8 (11.3%)	
Not available	8 (7.1%)	1 (2.4%)	7 (9.8%)	
**LN status**				
N0	52 (46%)	27 (64.3%)	0	
N1	25 (22.1%)	9 (21.4%)	28 (39.4%)	
N2	26 (23%)	4 (9.5%)	35 (49.3%)	
Not available	10 (8.9%)	2 (4.8%)	8 (11.3%)	
**Metastatic site**				
Lung	21 (18.6%)	0	21 (29.6%)	
Liver	55 (48.7%)	0	55 (77.5%)	
Pancreas	1 (0.9%)	0	1 (1.4%)	
Bone	1 (0.9%)	0	1 (1.4%)	
Peritoneum	10 (8.9%)	0	10 (14%)	
Brain	2 (1.8%)	0	2 (2.8%)	
Testis	1 (0.9%)	0	1 (1.4%)	
Uterus	1 (0.9%)	0	1 (1.4%)	

A: ascending; BMI: body mass index; C: cecum; D: descending; LN: lymph node; R: rectum; S: sigmoid; T = transverse.

**Table 3:** DMCs at the first exon or close to the TSS identified by in silico analysis in *CRFR1*.

STUDY	CpG ID	Compared Study Groups	Mean β-Value 1 *	Mean β-Value 2 *	Δ β-Value ^#^	Methylation, CRC vs. Normal	Gene Location	Location Relative to CpG	FDR
GSE149 282	cg08473090	Adjacent vs. CRC tissue	0.089	0.330	+0.241	Up	TSS1500	Island	3.174 × 10^−3^
GSE149 282	cg08929103	Adjacent vs. CRC tissue	0.563	0.239	−0.323	Down	TSS1500	N shore	4.453 × 10^−5^
GSE149 282	cg11338426	Adjacent vs. CRC tissue	0.103	0.269	+0.166	Up	First Exon	Island	1.786 × 10^−2^
GSE149 282	cg12577105	Adjacent vs. CRC tissue	0.047	0.163	+0.116	Up	TSS1500	Island	8.456 × 10^−3^
GSE149 282	cg13521908	Adjacent vs. CRC tissue	0.077	0.249	+0.172	Up	First Exon	Island	3.136 × 10^−3^
GSE149 282	cg18757974	Adjacent vs. CRC tissue	0.062	0.218	+0.156	Up	TSS1500	Island	7.654 × 10^−3^
GSE122 2126	cg08929103	Healthy vs. CRC ccfDNA	0.767	0.477	−0.291	Down	TSS1500	N shore	1.261 × 10^−3^
GSE122 2126	cg13521908	Healthy vs. CRC ccfDNA	0.117	0.213	+0.096	Up	First Exon	Island	2.545 × 10^−2^

* Mean β-value 1 represents methylation in normal tissues and mean β-value 2—methylation in diseased tissues; ^#^ Δ β-value: mean β-value 2 − mean β-value 1; DMC: differentially methylated CpG; FDR: false discovery rate; ccfDNA: circulating cell-free DNA.

**Table 4:** DMCs at the first exon or close to the TSS identified by in silico analysis in *CRFR2.*

STUDY	CpG ID	Compared Study Groups	Mean β-Value 1 *	Mean β-Value 2 *	Δ β-Value ^#^	Methylation, Diseased vs. Normal	Gene Location	Location Relative to CpG	FDR
GSE149 282	cg01718447	Adjacent vs. CRC tissue	0.126	0.536	+0.410	Up	TSS200	Island	2.375 × 10^−3^
GSE149 282	cg02712145	Adjacent vs. CRC tissue	0.166	0.471	+0.305	Up	TSS1500	Island	2.785 × 10^−2^
GSE149 282	cg04922810	Adjacent vs. CRC tissue	0.077	0.435	+0.358	Up	First Exon	Island	7.024 × 10^−3^
GSE149 282	cg07658503	Adjacent vs. CRC tissue	0.051	0.313	+0.262	Up	TSS200	Island	3.165 × 10^−2^
GSE149 282	cg13094036	Adjacent vs. CRC tissue	0.089	0.343	+0.254	Up	TSS1500	Island	3.663 × 10^−3^
GSE149 282	cg14896516	Adjacent vs. CRC tissue	0.106	0.352	+0.246	Up	TSS1500	Island	1.861 × 10^−4^
GSE149 282	cg18266052	Adjacent vs. CRC tissue	0.100	0.418	+0.318	Up	First Exon	Island	4.872 × 10^−2^
GSE149 282	cg21773872	Adjacent vs. CRC tissue	0.146	0.655	+0.509	Up	TSS200	Island	1.242 × 10^−6^
GSE149 282	cg24214442	Adjacent vs. CRC tissue	0.123	0.463	+0.340	Up	First Exon	Island	3.225 × 10^−6^
GSE149 282	cg24610236	Adjacent vs. CRC tissue	0.074	0.451	+0.378	Up	First Exon	Island	1.568 × 10^−5^
GSE149 282	cg27430726	Adjacent vs. CRC tissue	0.133	0.457	+0.325	Up	First Exon	Island	7.102 × 10^−5^
GSE1222126	cg04863452	Healthy vs. CRC ccfDNA	0.053	0.110	+0.057	Up	TSS200	Island	1.065 × 10^−3^
GSE1222126	cg04923928	Healthy vs. CRC ccfDNA	0.045	0.143	+0.098	Up	First Exon	Island	1.665 × 10^−4^
GSE1222126	cg15615793	Healthy vs. CRC ccfDNA	0.486	0.629	+0.142	Up	TSS1500	S_Shore	9.913 × 10^−3^
GSE1222126	cg18351440	Healthy vs. CRC ccfDNA	0.885	0.806	−0.079	Down	TSS1500	N_Shelf	3.929 × 10^−5^
GSE105799	cg21773872	Normal vs. CD	0.089	0.041	−0.049	Down	TSS200	Island	2.386 × 10^−4^

* Mean β-value 1 represents methylation in normal tissues and mean β-value 2—methylation in diseased tissues; ^#^ Δ β-value: mean β-value 2 − mean β-value 1. DMCs: differentially methylated CpGs; FDR: false discovery rate; ccfDNA: circulating cell-free DNA.

## Data Availability

Data are available upon request.

## Supplementary Materials

The following are available online at https://www.mdpi.com/article/10.3390/jcm10122680/s1, Table S1: DMCs identified by in silico analysis in *CRFR1* among the studied groups, Table S2: DMCs identified by in silico analysis in *CRFR2* among the studied groups.
