# Supplementary material for: Methylation Status of Corticotropin-Releasing Factor (CRF) Receptor Genes in Colorectal Cancer
Source: J Clin Med. 2021 Jun 18;10(12):2680. doi: 10.3390/jcm10122680 (PMC8234503; doi:10.3390/jcm10122680)
Supplement: Supplementary file 1 [file jcm-10-02680-s001.zip › supplementary Tables S1 and S2.pdf]

Table S1: DMCs identified by *in silico* analysis in *CRFR1* among studied groups.

STUDY	CG ID	Correlation	Mean β value 1*	Mean β value 2*	Δ β value#	Methylation in disease vs normal	Location relative to gene	Location relative to CpG	FDR
GSE149282	cg00025823	Adjacent vs CRC tissue	0.491	0.298	-0.193	Down	Gene body		7.209E-03
GSE149282	cg02810898	Adjacent vs CRC tissue	0.798	0.621	-0.177	Down	5'UTR		3.188E-02
GSE149282	cg03066966	Adjacent vs CRC tissue	0.591	0.335	-0.256	Down	5'UTR		1.003E-03
GSE149282	cg03323388	Adjacent vs CRC tissue	0.803	0.587	-0.217	Down	5'UTR		8.953E-04
GSE149282	cg07657976	Adjacent vs CRC tissue	0.825	0.483	-0.342	Down	5'UTR		2.459E-03
GSE149282	cg07778819	Adjacent vs CRC tissue	0.138	0.554	+0.416	Up	GB	Island	2.871E-07
GSE149282	cg00022871	Adjacent vs CRC tissue	0.637	0.349	-0.288	Down	GB		2.446E-04
GSE149282	cg09422970	Adjacent vs CRC tissue	0.492	0.163	-0.329	Down	5'UTR		3.674E-06
GSE149282	cg10106856	Adjacent vs CRC tissue	0.870	0.651	-0.219	Down	5'UTR		4.361E-02
GSE149282	cg10256584	Adjacent vs CRC tissue	0.770	0.626	-0.144	Down	5'UTR		5.594E-02
GSE149282	cg11731737	Adjacent vs CRC tissue	0.079	0.265	+0.186	Up	GB	Island	2.621E-02
GSE149282	cg14297797	Adjacent vs CRC tissue	0.381	0.147	-0.235	Down	5'UTR		5.261E-03
GSE149282	cg15117716	Adjacent vs CRC tissue	0.517	0.158	-0.359	Down	5'UTR		4.155E-06
GSE149282	cg15607306	Adjacent vs CRC tissue	0.913	0.725	-0.188	Down	GB		1.366E-03
GSE149282	cg16642545	Adjacent vs CRC tissue	0.614	0.357	-0.257	Down	GB		1.029E-03
GSE149282	cg16830379	Adjacent vs CRC tissue	0.745	0.554	-0.191	Down	3'UTR		8.893E-03
GSE149282	cg17311440	Adjacent vs CRC tissue	0.686	0.373	-0.313	Down	5'UTR		1.764E-05
GSE149282	cg27551605	Adjacent vs CRC tissue	0.088	0.330	+0.243	Up	GB	Island	1.541E-04
GSE149282	cg23420656	Adjacent vs CRC tissue	0.429	0.195	-0.234	Down	5'UTR	S_Shelf	4.167E-03
GSE149282	cg24394631	Adjacent vs CRC tissue	0.206	0.475	+0.269	Up	Gene body	S_Shore	2.585E-05
GSE149282	cg24738082	Adjacent vs CRC tissue	0.887	0.723	-0.165	Down	5'UTR		6.352E-03
GSE149282	cg26656751	Adjacent vs CRC tissue	0.678	0.391	-0.286	Down	GB		1.235E-04
GSE149282	cg27410679	Adjacent vs CRC tissue	0.496	0.153	-0.343	Down	GB	S_Shelf	6.879E-06
GSE149282	cg18757974	Adjacent vs CRC tissue	0.062	0.218	+0.156	Up	TSS1500	Island	7.654E-03
GSE149282	cg12577105	Adjacent vs CRC tissue	0.047	0.163	+0.116	Up	TSS1500	Island	8.456E-03
GSE149282	cg13521908	Adjacent vs CRC tissue	0.077	0.249	+0.172	Up	1st Exon	Island	3.136E-03
GSE149282	cg11338426	Adjacent vs	0.103	0.269	+0.166	Up	1st Exon	Island	1.786E-02

		CRC tissue							
GSE149282	cg08929103	Adjacent vs CRC tissue	0.563	0.239	-0.323	Down	TSS1500	N_Shore	4.453E-05
GSE149282	cg08473090	Adjacent vs CRC tissue	0.089	0.330	+0.241	Up	TSS1500	Island	3.174E-03
GSE1222126	cg00022871	Healthy vs CRC ccfDNA	0.875	0.670	-0.205	Down	GB		2.533E-03
GSE1222126	cg10256584	Healthy vs CRC ccfDNA	0.909	0.836	-0.073	Down	5'UTR		8.778E-03
GSE1222126	cg11524343	Healthy vs CRC ccfDNA	0.377	0.631	+0.254	Up	5'UTR		1.382E-02
GSE1222126	cg11760414	Healthy vs CRC ccfDNA	0.386	0.495	+0.109	Up	5'UTR	S_Shore	5.105E-02
GSE1222126	cg13947929	Healthy vs CRC ccfDNA	0.391	0.488	+0.097	Up	GB	S_Shore	2.432E-02
GSE1222126	cg15117716	Healthy vs CRC ccfDNA	0.855	0.418	-0.438	Down	5'UTR		1.166E-04
GSE1222126	cg15607306	Healthy vs CRC ccfDNA	0.941	0.889	-0.051	Down	GB		4.413E-02
GSE1222126	cg16642545	Healthy vs CRC ccfDNA	0.764	0.436	-0.328	Down	GB		1.991E-03
GSE1222126	cg16830379	Healthy vs CRC ccfDNA	0.875	0.715	-0.160	Down	3'UTR		1.130E-04
GSE1222126	cg24063856	Healthy vs CRC ccfDNA	0.231	0.390	+0.159	Up	GB	S_Shore	1.658E-03
GSE1222126	cg24738082	Healthy vs CRC ccfDNA	0.931	0.863	-0.068	Down	5'UTR		2.627E-03
GSE1222126	cg27410679	Healthy vs CRC ccfDNA	0.689	0.363	-0.326	Down	GB	S_Shelf	1.740E-03
GSE1222126	cg08929103	Healthy vs CRC ccfDNA	0.767	0.477	-0.291	Down	TSS1500	N_Shore	1.261E-03
GSE1222126	cg13521908	Healthy vs CRC ccfDNA	0.117	0.213	+0.096	Up	1st Exon	Island	2.545E-02
GSE105798	cg13947929	Normal vs CD	0.302	0.452	+0.150	Up	GB	S_Shore	1.581E-03
GSE105798	cg00025823	Normal vs CD	0.475	0.606	+0.130	Up	GB		9.794E-03
GSE105798	cg24063856	Normal vs CD	0.185	0.347	+0.162	Up	GB	S_Shore	5.160E-03

*Mean β value 1 represents methylation in normal and Mean β value 2 methylation in disease; #Δ β value: Mean β value 2-Mean β value 1. Open rows correspond to DMCs located at the gene body and shaded rows to DMCs located at the 1st Exon or close to TSS. GB: Gene Body; DMCs: Differentially methylated CpGs; CRC: Colorectal Cancer; FDR: False Discovery Rate; ccfDNA: circulating cell-free DNA; CD: Crohn's disease

Table S2: DMCs identified by *in silico* analysis in *CRFR2* among studied groups.

Study	CpG ID	Correlation	Mean β value 1*	Mean β value 2*	Δ β value#	Methylation in disease vs normal	Location relative to gene	Location relative to CpG	FDR
GSE149282	cg01049782	Adjacent vs CRC tissue	0.607	0.188	-0.418	Down	GB	N_Shelf	3.314E-02
GSE149282	cg01819552	Adjacent vs CRC tissue	0.658	0.348	-0.311	Down	GB		6.482E-02
GSE149282	cg03484834	Adjacent vs CRC tissue	0.650	0.461	-0.189	Down	GB		1.305E-02
GSE149282	cg03667083	Adjacent vs CRC tissue	0.622	0.775	+0.153	Up	GB	N_Shore	8.689E-02
GSE149282	cg05366813	Adjacent vs CRC tissue	0.580	0.287	-0.293	Down	GB	S_Shore	2.593E-02
GSE149282	cg05877083	Adjacent vs CRC tissue	0.807	0.475	-0.332	Down	GB		2.812E-02
GSE149282	cg06866646	Adjacent vs CRC tissue	0.840	0.500	-0.340	Down	GB		2.476E-02
GSE149282	cg09516959	Adjacent vs CRC tissue	0.238	0.530	+0.292	Down	GB	Island	1.463E-04
GSE149282	cg12511160	Adjacent vs CRC tissue	0.043	0.026	-0.017	Down	GB		5.222E-02
GSE149282	cg16127724	Adjacent vs CRC tissue	0.705	0.545	-0.159	Down	GB		1.089E-03
GSE149282	cg16755766	Adjacent vs CRC tissue	0.600	0.254	-0.346	Down	GB		1.128E-03
GSE149282	cg17924854	Adjacent vs CRC tissue	0.636	0.310	-0.326	Down	GB		4.340E-04
GSE149282	cg23185751	Adjacent vs CRC tissue	0.800	0.503	-0.297	Down	GB	N_Shore	2.140E-02
GSE149282	cg24430106	Adjacent vs CRC tissue	0.953	0.786	-0.166	Down	GB		1.194E-03
GSE149282	cg27191795	Adjacent vs CRC tissue	0.894	0.625	-0.269	Down	GB	N_Shelf	1.160E-04
GSE149282	cg01718447	Adjacent vs CRC tissue	0.126	0.536	+0.410	Up	TSS200	Island	2.375E-03
GSE149282	cg02712145	Adjacent vs CRC tissue	0.166	0.471	+0.305	Up	TSS1500	Island	2.785E-02
GSE149282	cg04922810	Adjacent vs CRC tissue	0.077	0.435	+0.358	Up	1st Exon	Island	7.024E-03
GSE149282	cg07658503	Adjacent vs CRC tissue	0.051	0.313	+0.262	Up	TSS200	Island	3.165E-02
GSE149282	cg13094036	Adjacent vs CRC tissue	0.089	0.343	+0.254	Up	TSS1500	Island	3.663E-03
GSE149282	cg14896516	Adjacent vs CRC tissue	0.106	0.352	+0.246	Up	TSS1500	Island	1.861E-04
GSE149282	cg18266052	Adjacent vs CRC tissue	0.100	0.418	+0.318	Up	1st Exon	Island	4.872E-02
GSE149282	cg21773872	Adjacent vs CRC tissue	0.146	0.655	+0.509	Up	TSS200	Island	1.242E-06
GSE149282	cg24214442	Adjacent vs CRC tissue	0.123	0.463	+0.340	Up	1st Exon	Island	3.225E-06
GSE149282	cg24610236	Adjacent vs CRC tissue	0.074	0.451	+0.378	Up	1st Exon	Island	1.568E-05
GSE149282	cg27430726	Adjacent vs CRC tissue	0.133	0.457	+0.325	Up	1st Exon	Island	7.102E-05

GSE1222126	cg16127724	Healthy vs CRC ccfDNA	0.884	0.676	-0.207	Down	GB		6.867E-05
GSE1222126	cg16755766	Healthy vs CRC ccfDNA	0.767	0.422	-0.345	Down	GB		2.801E-06
GSE1222126	cg01819552	Healthy vs CRC ccfDNA	0.813	0.585	-0.228	Down	GB		6.859E-04
GSE1222126	cg01049782	Healthy vs CRC ccfDNA	0.778	0.327	-0.451	Up	GB	N_Shelf	5.024E-03
GSE1222126	cg01972879	Healthy vs CRC ccfDNA	0.797	0.887	+0.090	Up	GB	N_Shore	2.583E-04
GSE1222126	cg03667083	Healthy vs CRC ccfDNA	0.583	0.757	+0.174	Up	GB	N_Shore	2.023E-02
GSE1222126	cg05366813	Healthy vs CRC ccfDNA	0.850	0.643	-0.207	Down	GB	S_Shore	2.511E-03
GSE1222126	cg22826063	Healthy vs CRC ccfDNA	0.859	0.714	-0.145	Down	GB		2.428E-02
GSE1222126	cg06175988	Healthy vs CRC ccfDNA	0.686	0.479	-0.208	Down	GB	N_Shelf	4.417E-02
GSE1222126	cg27191795	Healthy vs CRC ccfDNA	0.934	0.755	-0.178	Down	GB	N_Shelf	8.228E-07
GSE1222126	cg04863452	Healthy vs CRC ccfDNA	0.053	0.110	+0.057	Up	TSS200	Island	1.065E-03
GSE1222126	cg18351440	Healthy vs CRC ccfDNA	0.885	0.806	-0.079	Down	TSS1500	N_Shelf	3.929E-05
GSE1222126	cg04923928	Healthy vs CRC ccfDNA	0.045	0.143	+0.098	Up	1st Exon	Island	1.665E-04
GSE1222126	cg15615793	Healthy vs CRC ccfDNA	0.486	0.629	+0.142	Up	TSS1500	S_Shore	9.913E-03
GSE105798	cg01972879	Normal vs CD	0.869	0.779	-0.089	Down	GB	N_Shore	2.641E-03
GSE105798	cg21773872	Normal vs CD	0.089	0.041	-0.049	Down	TSS200	Island	2.386E-04

*Mean β value 1 represents methylation in normal and Mean β value 2 represents methylation in disease; #Δ β value: Mean β value 2-Mean β value 1. Open rows correspond to DMCs located at the gene body and shaded rows to DMCs located at the 1st Exon or close to TSS. GB: Gene Body; DMCs: Differentially methylated CpGs; CRC: Colorectal Cancer; FDR:False Discovery Rate; ccfDNA: circulating cell-free DNA; CD: Crohn's disease.
